# Supplementary material for: The in vivo hydrocarbon formation by vanadium nitrogenase follows a secondary metabolic pathway
Source: Nat Commun. 2016 Dec 15;7:13641. doi: 10.1038/ncomms13641 (PMC5172229; doi:10.1038/ncomms13641)
Supplement: Supplementary Information — Supplementary Figure 1 [file ncomms13641-s1.pdf]

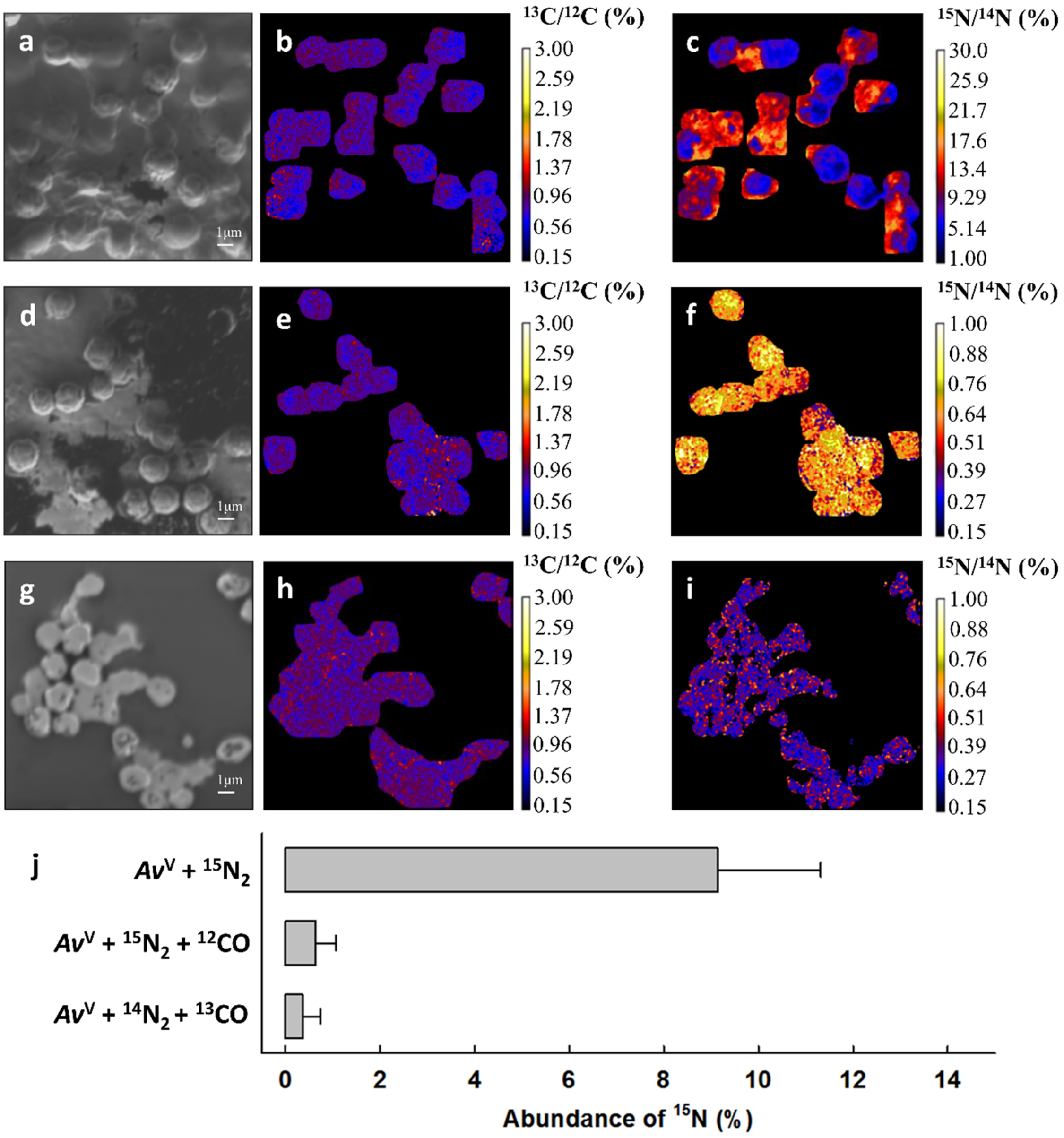

Supplementary Figure 1 | Assimilation of N via N_2 reduction by *A. vinelandii*. (**a-i**) Secondary electron (**a, d, g**) and secondary ion (**b-h, c-i**) images derived from nanoSIMS analysis of *A. vinelandii* cells expressing the V-nitrogenase in the presence of $^{15}N_2$ (**a-c**), $^{15}N_2$ plus ^{12}CO (**d-e**), and $^{14}N_2$ plus ^{13}CO (**g-i**) (see Methods section for details of experiments). For each sample, data were collected at 3 different regions of interest (ROI), 2 frames/ROI. Representative nanoSIMS data are shown in a-i. (**j**) Average ^{15}N abundance ($^{15}N/^{14}N$ ratio) of *A. vinelandii* cells expressing the V-nitrogenase in the presence of $^{15}N_2$, $^{15}N_2$ plus ^{12}CO, and $^{15}N_2$ plus ^{13}CO. The average ^{15}N abundance of each sample was calculated based on data collected in 3 different ROIs ($n = 6$). Scale bars representing 1 µm are shown in a, d and g.
